# Up-regulation of antioxidative proteins TRX1, TXNL1 and TXNRD1 in the cortex of PTZ kindling seizure model mice

**DOI:** 10.1371/journal.pone.0210670

**Published:** 2019-01-24

**Authors:** Jia-Tian Yu, Ye Liu, Ping Dong, Run-En Cheng, Shao-Xi Ke, Kai-Qin Chen, Jing-Jing Wang, Zhong-Shan Shen, Qiong-Yao Tang, Zhe Zhang

**Affiliations:** 1 Jiangsu Province Key Laboratory of Anesthesiology, Xuzhou Medical University, Xuzhou, Jiangsu Province, China; 2 Jiangsu Province Key Laboratory of Anesthesia and Analgesia Application Technology, Xuzhou Medical University, Xuzhou, Jiangsu Province, China; 3 Department of Anatomy, College of Biomedical Sciences, Xuzhou Medical University, Xuzhou, Jiangsu Province, China; 4 School of Clinical Medicine, Xuzhou Medical University, Xuzhou, Jiangsu Province, China; Virginia Commonwealth University, UNITED STATES

## Abstract

Oxidative stress has been considered as one of pathogenesis of brain damage led by epilepsy. Reducing oxidative stress can ameliorate brain damage during seizures. However, expression levels of important antioxidative enzymes such as thioredoxin-1 (TRX1), thioredoxin-like 1 protein (TXNL1) and thioredoxin reductase 1 (TXNRD1) during seizures have not been investigated. In this study, we examined protein and mRNA expression levels of TRX1, TXNL1 and TXNRD1 in different brain regions in PTZ induced seizure model mice. We found that protein expression levels of TRX1, TXNL1 and TXNRD1 are simultaneously up-regulated by 2- or 3-fold in the cortex of both acute and chronic seizure model mice. But there is no unified expression pattern change of these enzymes in the hippocampus, cerebellum and diencephalon in the seizure model mice. Less extent up-regulation of mRNA expression of these enzymes were also observed in the cortex of seizure mice. These data suggest that antioxidative enzymes may provide a protective effect against oxidative stress in the cortex during seizures.

## Introduction

Epileptic seizure is excessive and abnormal neuronal activity from a specific region of the brain, which may lead to convulsion, decreased level of consciousness, and other sensory or motor symptoms. Causes of epilepsy include all kinds of genetic ion channel mutations, altered cortical development and specific gene expression changes [1–4]. Frequent seizures may lead to neurodegeneration and generate oxidative stress by hyper-excitability. During abnormal excitable neuronal synaptic release process, activation of ionotropic glutamate receptor triggers elevated intracellular Ca^2+^ entry at cellular level in the nervous system. As a consequence, high levels of intracellular calcium increase production of reactive oxygen species (ROS), which may result in toxic effects to neurons by producing peroxides and free radicals that damage all components of the cell, including proteins, lipids, and DNA [5–8].

In studies of examining the oxidative stress status in the brain in seizure models, activities of many oxidative stress related enzymes during seizures were examined, such as cytosolic Cu/Zn superoxide dismutase (SOD-1), mitochondrial Mn superoxide dismutase (SOD-2), and glutathione peroxidase (GPx), etc [9–11]. However, the expression levels of some thiol redox proteins, which serve as important antioxidative proteins during seizure, have not been examined. Among these thiol redox proteins, thioredoxin-1 (TRX1), thioredoxin-like protein 1 (TXNL1), and thioredoxin reductase 1 (TXNRD1) are richly expressed in the nervous system of mammalians [12–14]. The distribution of these proteins in the brain has also been studied. They are widely expressed in hippocampus, cortex and diencephalon (www.proteinatlas.org) [15, 16]. TRX1 is a small redox protein that is present in all organisms. It acts as an antioxidative protein by facilitating the reduction of other proteins due to the presence of two vicinal) cysteines in a CXXC motif. The TRX1 protein plays important role in reducing oxidative stress in the brain, a tissue that is prone to oxidative stress due to its high-energy demand [12, 17]. Thioredoxin reductase 1 (TXNRD1), a protein belongs to the family of pyridine nucleotide oxidoreductases, is another important protein for maintaining normal thiol redox state in the nervous system. This protein keeps TRX1 as well as other substrates in reducing state so that they play a vital role in prevention of oxidative stress. A study using mice with nervous system specific (NS-specific) deletion of TXNRD1 and TXNRD2 demonstrated while NS-specific TXNRD2 null mice develop normally, mice lacking TXNRD1 in the NS are significantly smaller and display ataxia and tremor [18, 19]. Thioredoxin-1 like protein 1 (TXNL1) is also a reductase that is involved in proteolysis, reducing oxidative phosphatase and promoting oxidative stress resistance [20–22]. Thus, expressing levels of these three reductases are critical for estimation the oxidative damage to the brain during seizures.

In this study, we examined the alteration of protein expression levels of these three enzymes in the different brain regions in PTZ induced adult seizure mice by western blot analysis. We also examined the mRNA expression levels of these enzymes in the different brain regions by using RT-PCR. Our results showed that proteins and mRNA expression levels of TRX1, TXNL1 and TXNR1 are up-regulated in the cortex in the PTZ kindled seizure mice. However, there is no unified expression level change of these enzymes in other brain regions that we examined. These results suggest these reductases play protective roles in the cortex of epileptic brain. Also, different brain regions have distinct oxidative stress status during seizures.

## Results

### Development of acute and chronic seizures following administration of PTZ

**Fig 1:**
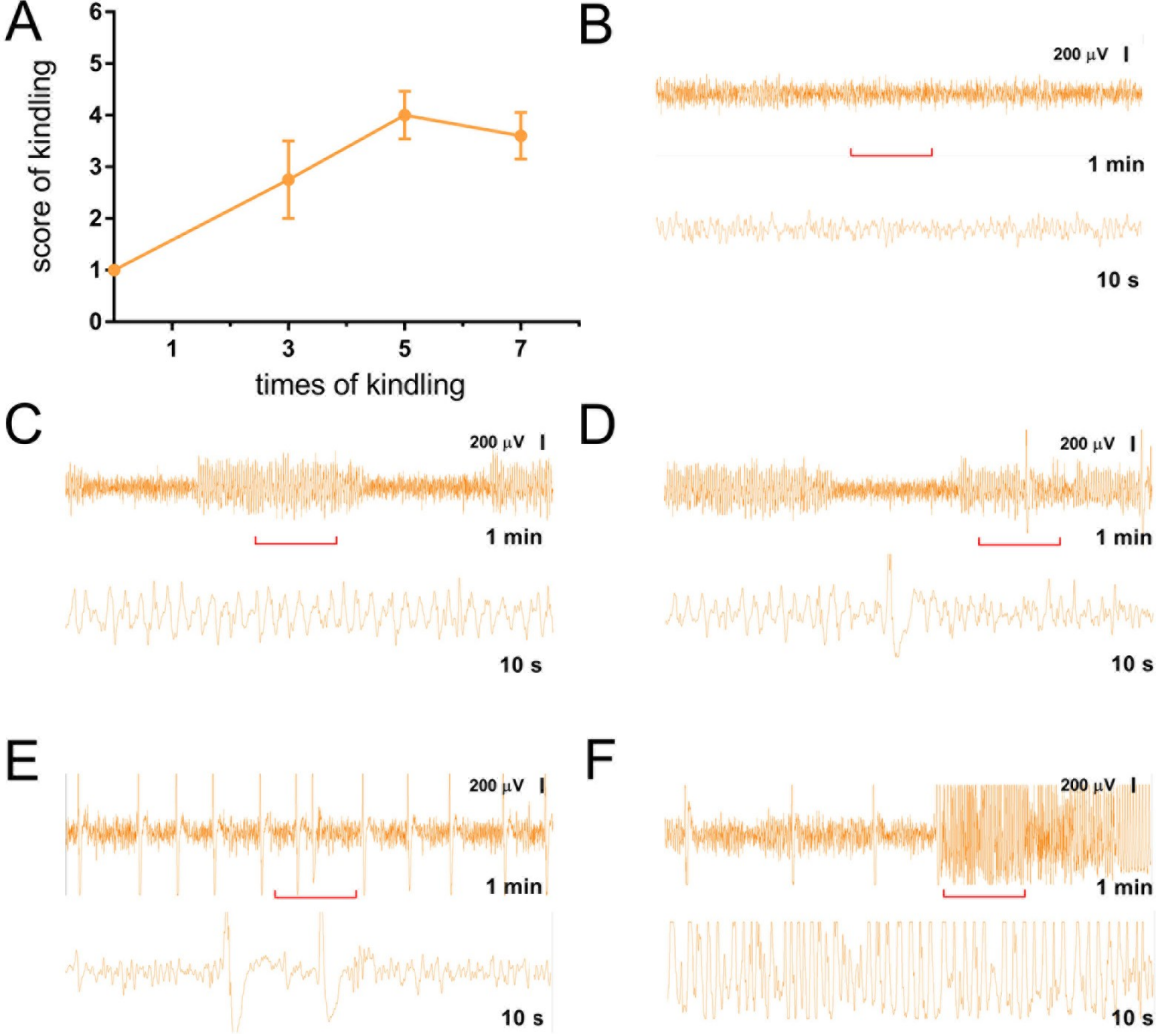
Electroencephalography (EEG) recordings and seizure behavior of mice before and after intraperitoneal injection of PTZ. **(A)** The relationship between score of seizures and times of injection in the chronic model mice were plotted. Injection was performed every other day. (**B)** Typical 1 min EEG trace of control mice. The red line lebeles10s piece of trace that is magnified and shown in down panel. Normal trace wave amplitude is less than 200 μV (**C-F)** Typical 1 min EEG traces of chronic model mice after injection of PTZ 3–7 times (top panel). The red lines indicate magnified 10s piece of traces that is shown in down panel. (**C)** 1 min EEG trace after 3 times of injection, correspond to sudden behavioral arrest and/or motionless staring. The biggest wave amplitude is larger than 200 μV (1st stage). The frequency of big spikes is about 3-6Hz. (**D)** Sample traces correspond to facial jerking with muzzle or eye and neck jerks (2nd stage) The biggest spike is more than 300 μV. (**E)** Typical traces that correspond to clonic seizure in a sitting position (3rd stage). The biggest wave amplitude is larger than 400 μV. (**F)** Typical traces correspond to convulsions including clonic seizures while lying on the belly and/or pure tonic seizures (4th stage). Amplitude of most spikes is more than 400 μV.

For generating acute seizure model mice, intraperitoneal injection of 70 mg/kg PTZ usually induces clonic convulsion of the whole body of the mouse that was injected. The intensity reached the level of 6th stage within 2–3 minutes [23]. Thus, it is not necessary to record EEG of these model mice. The mice were sacrificed 60 minutes after clonic seizure had reached 6th stage. The brain tissue was collected for RT-PCR and Western Blot analysis. For generating chronic seizure model, intraperitoneal injections of 35 mg/kg PTZ were repeated every other day. Their behavior and EEG were monitored after each injection for calculating seizure scale and confirming hyperactivity in the brain. As shown in Fig 1A, seizure score reached 2nd-3rd stage after 3 times injection according to revised Racine scale. Clonic seizure can be observed after 5–7 times (usually two weeks) injection (Fig 1A). In the meantime, EEG of these mice are recorded after PTZ injection. When normal mice are at an awake and quiet state, the normal EEG wave amplitude of mice is between 30–200 μV with frequency mainly from 6–13 Hz. No sharp wave or big spike was observed (Fig 1B). As indicated in Fig 1C, the abnormal EEG spike-wave discharges, containing 5 to 10 Hz big spikes with amplitude more than 200 μV, usually accompanied by the behavior of whisker trembling, arrest that corresponded to 1st stage of seizure, were observed after 3 doses of PTZ injections (Fig 1C). The second EEG abnormality, 4 to 6 Hz spike wave discharges, corresponded to sudden behavioral arrest and/or motionless staring of the 2nd stage seizure behavior, were also observed after 3 doses of PTZ injections (Fig 1D). After 5–7 doses of PTZ injection, the third EEG discharge pattern that contains 1–2 Hz big spikes with amplitude more than 800 μV, was found to coincide with the behavioral 3^rd^ stage seizure behavior such as facial jerking with muzzle or muzzle and eye (Fig 1E). The clonic seizures above 4^th^ stages accompanied by continued big spikes or waves with high amplitude more than 800 μV in EEG were also observed after 5–7 doses of injection (Fig 1F). The model mice were sacrificed to get brain tissues after 3, 5 and 7 doses of PTZ injection respectively.

### Protein expression level of TRX1, TXNL1 and TXNRD1 were up-regulated in the cortex of PTZ induced acute and chronic seizure model mouse

**Fig 2:**
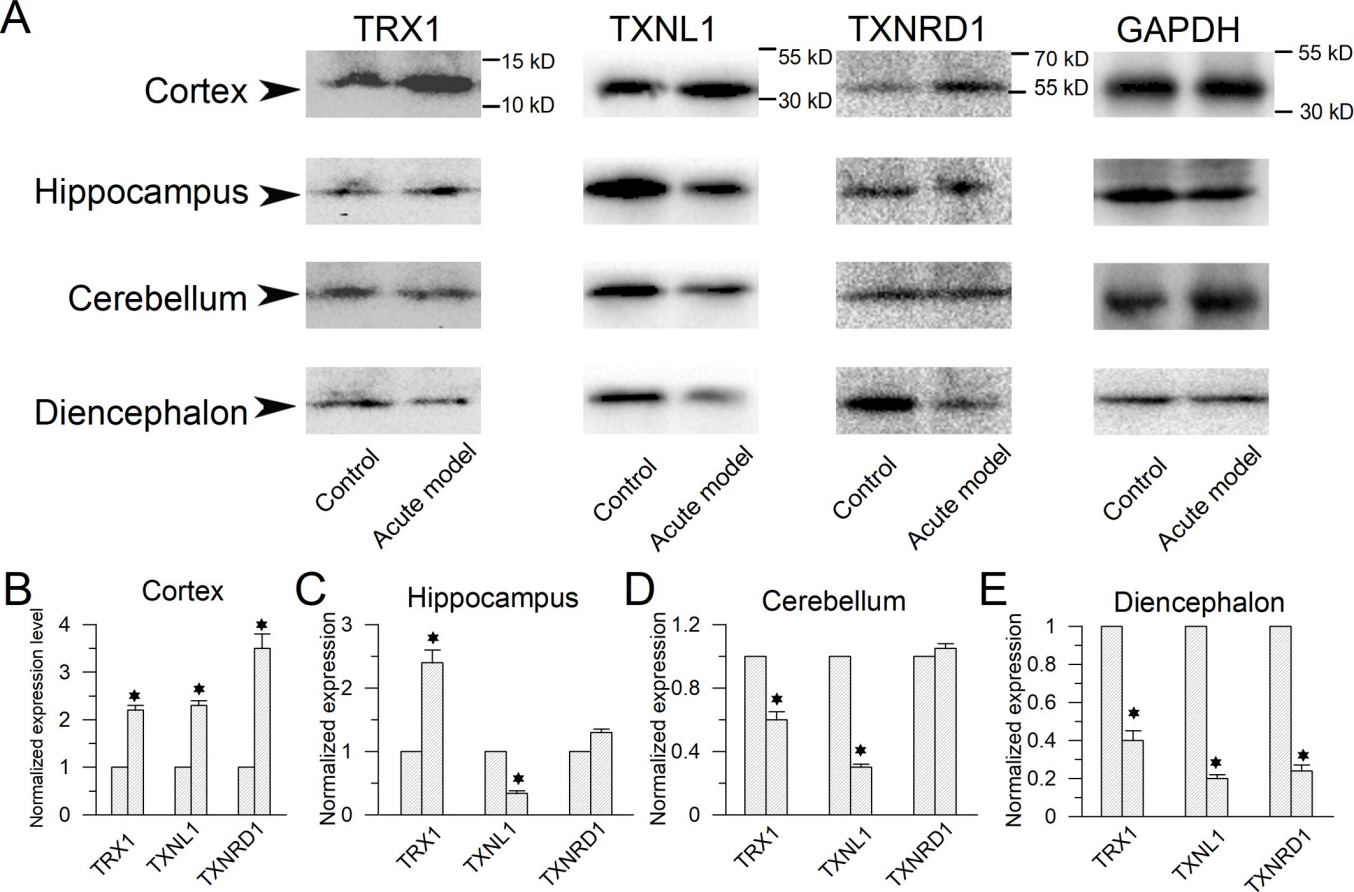
Protein expression level changes of TRX1, TXNL1 and TXNRD1 in PTZ induced acute epilepsy mice model. (**A)** Western blot analysis was performed on equal amount total proteins extracted from the cortex, hippocampus, cerebellum and diencephalon of PTZ induced seizure model mice and probed with the indicated antibodies. (**B-E)** The relative expression levels of protein in the cortex, hippocampus, cerebellum and diencephalon were determined by using gel-doc image lab system. The protein expression levels in different brain regions of control mice was set as basic value 1. (**B)** In the cortex, TRX1, TXNL1 and TXNRD1 protein expression levels were up-regulated by 2.2 ± 0.1, 2.3 ± 0.1and 3.5 ± 0.3 fold respectively (T test, * shows P<0.05). (**C)** In the hippocampus, protein expression levels of TRX1 and TXNRD1 are up-regulated 2.4 and 1.3 fold respectively (P < 0.05). The TXNL1 protein expression level was down-regulated to 34 ± 4% expression level of control (P<0.05). (**D)** In the cerebellum, the protein expression levels of TRX1, TXNL1 were down-regulated to 60% and 30% expression level of control (P<0.05). The TXNRD1 expression level is not altered. (**E)** In the Diencephalon, the protein expression of TRX1, TXNL1 and TXNRD1 are down-regulated to 40 ± 4%, 20 ± 2% and 24 ± 3% of the control levels respectively.

Since the TRX1, TXNL1 and TXNRD1 may reflect the oxidative stress level in the brain of seizure model, we examined protein expression levels of TRX1, TXNL1 and TXNRD1 by western blot analysis. The results showed that protein levels of TRX1, TXNL1and TXNRD1 were simultaneously up-regulated 2.3-, 2.4- and 3.5-fold respectively in the cortex of the *acute seizure* model mice (Fig 2A first line, Fig 2B left panel). However, there was no simultaneous up-regulation of these three enzymes in other brain regions. In the hippocampus of model mice, protein expression level of TRX1 was increased 2.5-fold while the TXNL1 protein level was decreased 2-fold. But the TXNRD1 expression level was not altered (Fig 2A second line, Fig 2C). In the cerebellum, TRX1 and TXNL1 protein expression levels were down-regulated but the TXNRD1 protein expression level was not altered (Fig 2A third line, Fig 2D). However, in the diencephalon, TRX1, TXNL1 and TXNRD1 proteins were all down-regulated (Fig 2A four line, Fig 2E). These results suggest that the oxidative stress in the cortex may trigger a compensatory antioxidative response of up-regulating proteins that are against oxidative stress. However, in other brain regions, either the oxidative stress may be not as strong as in the cortex or the antioxidative compensatory response is suppressed.

**Fig 3:**
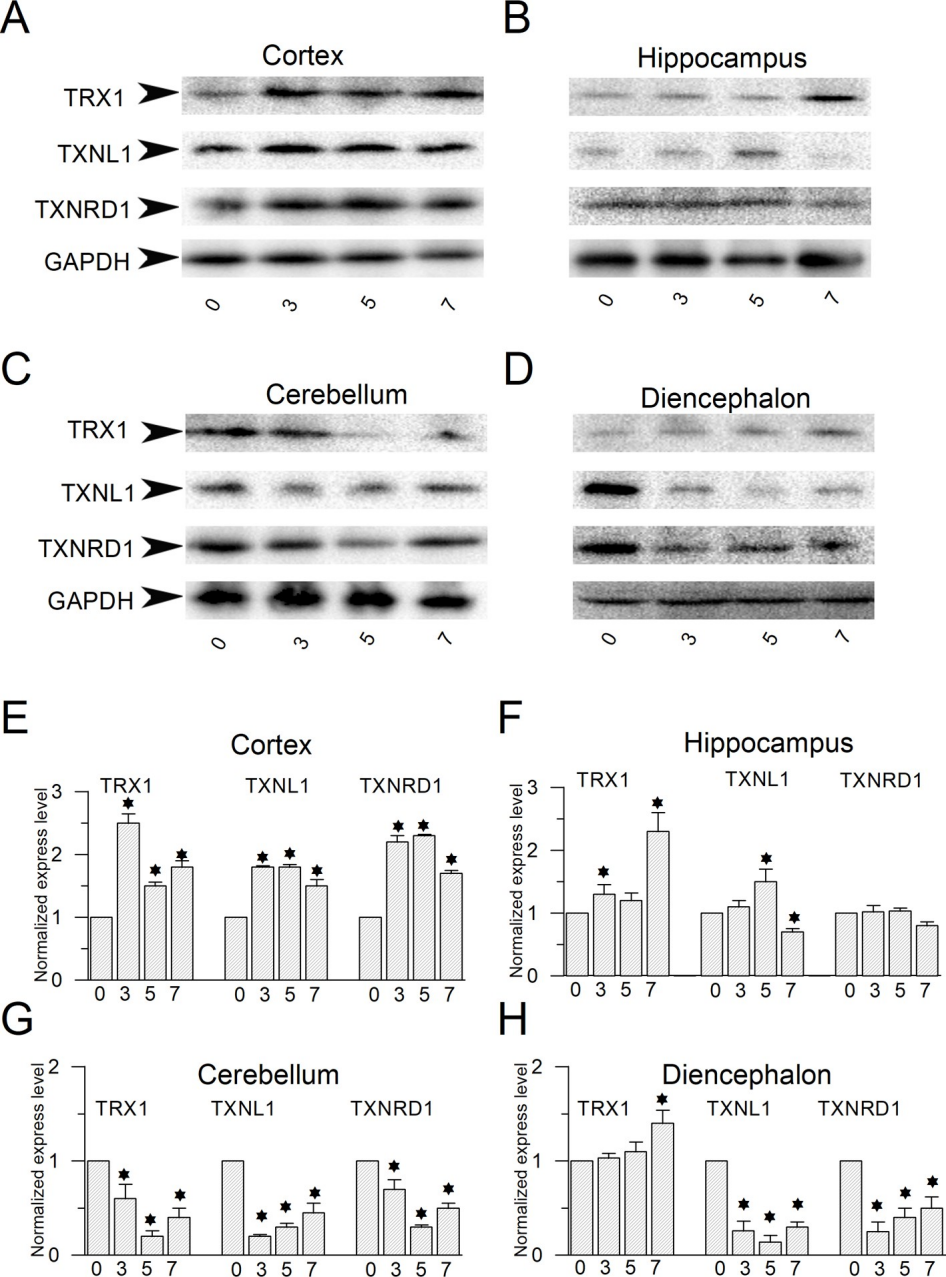
Protein expression level changes of TRX1, TXNL1 and TXNRD1 in PTZ induced chronic epilepsy mice model. (**A-D)** Western blot analysis of TRX1, TXNL1 and TXNRD1 protein levels in the cortex (A), the hippocampus (B), the cerebellum (C) and the diencephalon (D) of adult mice for indicated days after PTZ injection (n = 3). (**E-H**) Statistical analysis of protein expression levels in the cortex (E), hippocampus (F), cerebellum (G) and diencephalon (H) of adult mice before and on indicated days after PTZ injection. Protein bands were quantified by densitometry and normalized to the level of GAPDH. Data represent mean ± SEM. *P < 0.05. **(E)** In the cortex of chronic epileptic model mice after 3, 5 and 7 times of kindling, TRX1 protein expression levels were up-regulated by 2.5 ± 0.3, 1.5 ± 0.2 and 1.8 ± 0.25 fold respectively. Protein expression of TXNL1 were up-regulated by 1.8 ± 0.2, 1.8 ± 0.3 and 1.5 ± 0.2 fold respectively. Protein expression of TXNRD1 were up-regulated by 2.2 ± 0.2, 2.3 ± 0.2, 1.7 ± 0.2 respectively. (**F)** In the hippocampus of chronic epileptic model mice after 3, 5 and 7 times of kindling, TRX1 protein expression levels were up-regulated by 1.3 ± 0.15, 1.2 ± 0.18 and 1.8 ± 0.3 fold respectively. TXNL protein expression level was firstly up-regulated by 1.5 fold after 5 times of kindling but subsequently was down-regulated to 70% after 7 times of kindling. The TXNRD protein expression was not altered during the whole kindling process. (**G)** In the cerebellum of chronic epileptic model after 3, 5 and 7 times of kindling, the expression of TRX1, TXNL1 and TXNRD1 proteins are down-regulated. The TRX1 protein levels are (of the control level): 60 ± 15%, 20 ± 6% and 40 ± 10% respectively. The TXNL1 protein levels are 20 ± 2%, 30 ± 4% and 45 ± 10% respectively. TXNRD1 protein expression levels are 70 ± 10%, 30 ± 2% and 50 ± 5% of the control level respectively. (**H)** In the diencephalon after 3, 5 and 7 times of kindling, the normalized expression levels of TRX1 are (of the control level): 103 ± 0.05%, 110 ± 10% and 140 ± 14% respectively. The normalized expression levels of TXNL1 are (of the control level) 26 ± 10%, 14 ± 7% and 30 ± 5% respectively. The normalized expression levels of TXNRD1 are (of the control level) 25 ± 10%, 40 ± 10% and 50 ± 12% respectively.

In order to further study antioxidative roles of these enzymes are played in chronic seizures, we also examined the protein expression levels of these enzymes in different brain regions in chronic seizure model mouse brain induced by PTZ. Consistent with the result of acute seizure model, up-regulation of these enzymes in the cortex of chronic epileptic mouse model was observed after 3, 5 and 7 doses injection by PTZ. TRX1 protein expression levels were up-regulated 2.5-fold, 1.9-fold and 1.8-fold respectively (Fig 3A and 3E left). Protein expression levels of TXNL1 were up-regulated by 1.8-fold, 1.9-fold and 1.5-fold respectively (Fig 3A and 3E middle). Similarly, protein expression levels of TXNRD1 were up-regulated by 2.5-fold, 2.4-fold and 1.8-fold respectively (Fig 3A and 3E right). This data indicated PTZ could induce up-regulation of these enzymes in the cortex of chronic seizure model mice but the extent of up-regulation seemed not related to the score of kindling and doses of injection. However, in the hippocampus, TRX1 protein expression levels were not significantly altered at 3 doses and 5 doses PTZ injection but was elevated by 2.3-fold after 7 doses injection (Fig 3B). Moreover, the protein expression level of TXNL1 was not altered at 3 doses injection while the expression level was elevated 1.5-fold after 5 doses PTZ injection. However, the expression level of TXNL1 was decreased to 40% of the control level after 7 doses injection (Fig 3B). The protein expression levels of TXNRD1 in the hippocampus were also not altered during the entire kindling process (Fig 3B). This piece of data indicated that there is no strong antioxidative response in the hippocampus of chronic epileptic model mice. Interestingly, in the cerebellum, all these enzymes were down-regulated after 3–7 doses PTZ injection although the extent of down-regulation showed somewhat difference (Fig 3C and 3G). Similarly, down-regulation of expression levels of these three proteins were also observed in the diencephalon except a weak up-regulation of TRX1 after 7 doses of injection (Fig 3D and 3H). These results suggest that the antioxidative response to oxidative stress probably are also weak in the cerebellum and diencephalon.

### mRNA expression levels of TRX1, TXNL1 and TXNRD1 are up-regulated in the cortex of PTZ induced acute and chronic seizure model mouse

**Fig 4:**
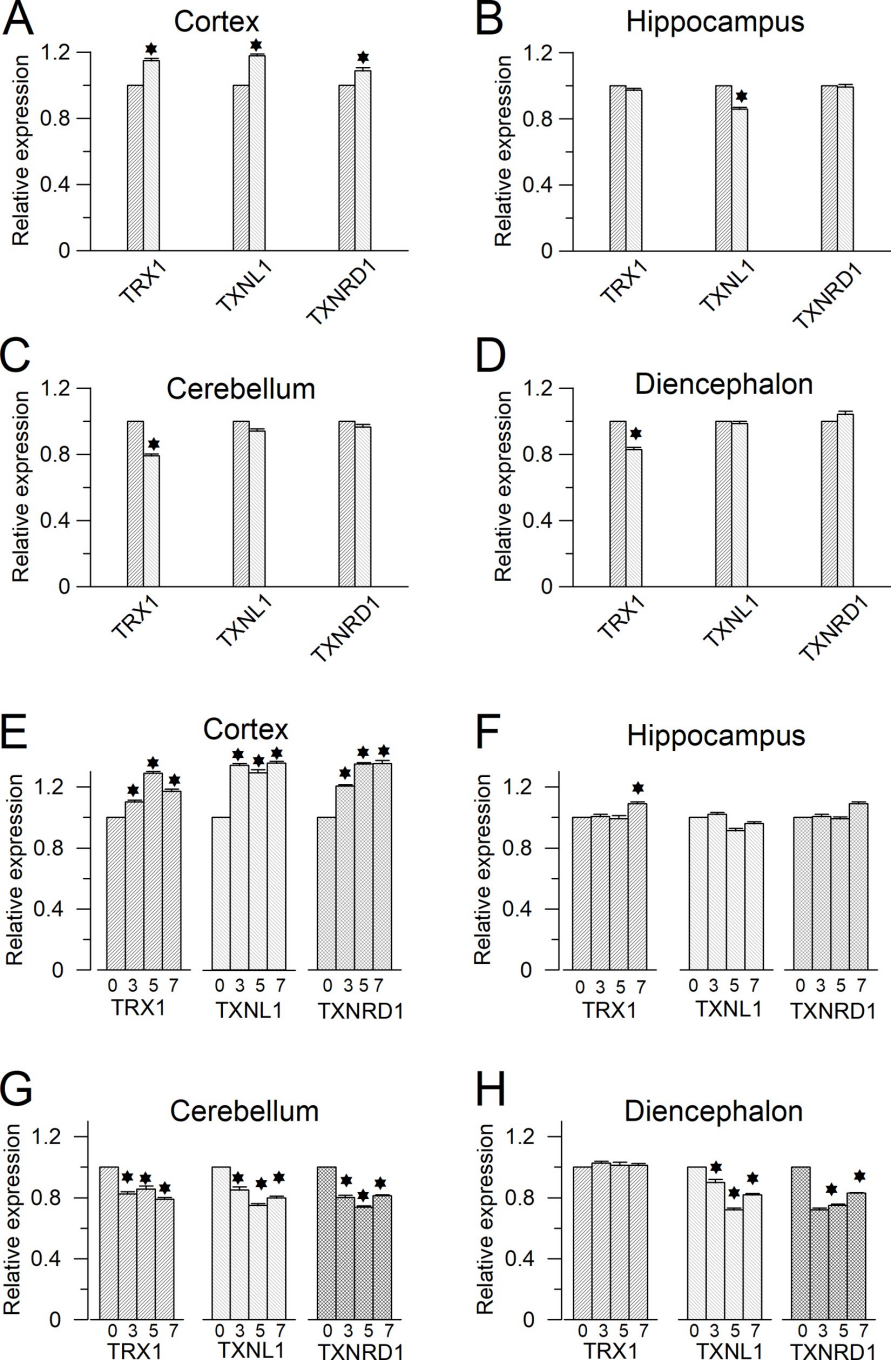
Seizure effect on mRNA expression of TRX1, TXNL1 and TXNRD1 subunit expression. **(A-D)** Relative mRNA expression levels of TRX1, TXNL1 and TXNRD1 in different brain regions of PTZ induced acute epilepsy adult mouse model (of the control). (**A)** Cortex, TRX1:114 ± 3%, TXNL1: 117% ± 1% and TXNRD1 108 ± 1%. (**B)** Hippocampus, TRX1: 97 ± 2%, TXNL1: 85 ± 1.5% and TXNRD1 99 ± 1.2%. (**C)** Cerebellum, TRX1: 79 ± 1.5%, TXNL1: 94 ± 1.4% and TXNRD1 97 ± 1.3%. (**D)** Diencephalon, TRX1: 83 ± 1.3%, TXNL1: 98 ± 1.2% and 104 ± 2% (**E-H)** Relative mRNA expression levels of TRX1, TXNL1 and TXNRD1 in the chronic model after 3, 5 and 7 times of kindling. (**E)** In the cortex, the normalized mRNA expression levels of TRX1 are: 110 ± 1.2%, 129 ± 1.1% and 117 ± 2%; TXNL1 are: 134 ± 1.3%, 129 ± 1.2% and 135 ± 1.5%; TXNRD1 are120 ± 1.4%, 135 ± 1.3% and 1.35 ± 2%. (**F)** In the hippocampus, the normalized mRNA expression levels of TRX1 are: 92 ± 1.3%, 89 ± 1.2% and 96 ± 1.2%; TXNL1 are: 102 ± 1.4%, 91 ± 2% and 96 ± 1.3%; TXNRD1 are100 ± 1.4%, 99 ± 1.3% and 108 ± 1.2%. (**G)** In the cerebellum, the normalized mRNA expression levels of TRX1 are: 82 ± 1.2%, 85 ± 1.1% and 79 ± 2%; TXNL1 are: 85 ± 2%, 75 ± 1.5% and 80 ± 1.2%; TXNRD1 are 80 ± 1.4%, 73 ± 1.3% and 81 ± 1.2%. (**H)** In the diencephalon, the normalized mRNA expression levels of TRX1 are (of the control): 90 ± 1.2%, 72 ± 1.1% and 81 ± 2%; TXNL1 are: 102 ± 2%, 101 ± 1.5% and 101 ± 1.2%; TXNRD1 are 72 ± 1.4%, 75 ± 1.3% and 83 ± 1.2%.

In order to further investigate the mechanism of protein expression level changes of TRX1, TXNL1 and TXNRD1, we further performed RT-PCR to examine the mRNA expression level changes of these enzymes. Consistent with the protein expression pattern changes of these enzymes, the mRNA expression levels of TRX1, TXNL1 and TXNRD1 were also up-regulated in the cortex in both the acute and the chronic seizure model mice (Fig 4A and 4E). However, the extent of up-regulation of mRNA expression levels of these enzymes is much less than the extent of protein expression level changes of these enzymes. In the acute epileptic model, the mRNA of TRX1, TXNL1 and TXNRD1 were up-regulated to 117%, 115% and 123% of the mRNA level of these enzymes in the normal mice cortex respectively (Fig 4A). Similarly, in the chronic model, all mRNA of these enzymes were only up-regulated by 15–30% percent after 3–7 doses PTZ injection (Fig 4E). These results indicated that the regulation mechanism of expression of these enzymes is mainly at translational level.

However, similar to protein expression pattern changes of these enzymes, there was no unified mRNA expression pattern change of these enzymes in other brain regions. In the hippocampus of acute epileptic model mice, the mRNA expression of TRX1 and TXNRD1 was not altered while mRNA expression of TXNL1 was somewhat down-regulated (Fig 4B). In the hippocampus of chronic epileptic model mice, the mRNA expression level of TRX1 was not altered after 3–5 doses of PTZ injection, but 20% increase of TRX1 mRNA expression was observed after 7 doses of PTZ injection. In the hippocampus, 15–25% decrease of mRNA expression of TXNL1 was observed during the whole injection process. The TXNRD1 mRNA expression level remained as same as the control expression level (Fig 4F). In summary, no strong increase of mRNA expression of these enzymes was observed in the hippocampus. This fact reflects again that a weak antioxidative response in the hippocampus of the seizure model mouse.

In the cerebellum of both acute and chronic epileptic model mice, mRNA expression levels of TRX1 were decreased by 20% (Fig 4C). However, mRNA expression levels of TXNL1 and TXNRD1 were not altered in the cerebellum in acute seizure model mice. But mRNA expression levels of TXNL1 and TXNRD1 were down-regulated in the cerebellum of chronic seizure model mice (Fig 4C and 4G). In the diencephalon, 20% percent decrease of TRX1 mRNA was observed in acute seizure model mice. But mRNA expression levels of TRX1 were not altered in the diencephalon of chronic seizure model mice (Fig 4D and 4H). The mRNA expression levels of TXNL1 and TXNRD1 were not altered in the diencephalon of acute seizure model mice (Fig 4D). But in the diencephalon of chronic seizure model mice, the mRNA express levels of TXNL1 and TXNRD1 were down-regulated by 10–20% (Fig 4H). These data also indicated that there is no obvious antioxidative response in the cerebellum and diencephalon of PTZ kindled mice.

## Discussion

In this study, we present data showing protein and mRNA of important reductases are distinctly regulated in different brain regions in PTZ induced acute and chronic seizure model mice. These data for the first time provide biochemical evidences to show that oxidative stress states in different brain regions of PTZ kindling seizure model are distinct. As we observed, the antioxidative response reflected by up-regulation of TRX1, TXNL1 and TXNRD1 mainly occurred in the cortex and somewhat in the hippocampus. Furthermore, the down-regulation of TRX1, TXNRD1 in the cerebellum and diencephalon either reflects that less oxidative stress happened or absence of antioxidative response in these brain regions. Previous observation of EPR (electron paramagnetic resonance) imaging in the brain of PTZ kindling model mice indicated that strong oxidative stress occurs in the cortex and hippocampus but relative weak oxidative stress is produced in the cerebellum [24, 25]. Thus, the best explanation of the down-regulation of thiol redox protein expression in the cerebellum is relatively weak oxidative stress produced in the cerebellum. Since the up-regulation of antioxidative enzymes expression may ameliorate the oxidative damage to the cortex, these data also suggest that the hippocampus may be more vulnerable to oxidative damage than the cortex.

Oxidative stress damage to the epileptic brain includes increased amount of free radicals, excessive generation of NO^-^ and dysfunction of mitochondria [5, 26]. Although the brain is vulnerable to oxidative stress due to its high oxygen utilization, production of ROS may activate defense system existed in the brain against the effects of these oxidative species during seizures. For example, Mn^2+^ superoxide dismutase (Mn-SOD), one of endogenous radical defense enzyme, is up-regulated in chronic PTZ kindled rat seizure model [27]. However, the activity of total SOD and level of another endogenous antioxidant α-tocopherol are significantly decreased in acute PTZ kindling model [27]. Also, thiol redox status (TRS) components such as glutathione (GSH), glutathione disulfide (GSSG), cysteine (CSH), protein (P) thiols (PSH) are all down-regulated in the cerebral cortex of PTZ induced seizure mice [28]. But these data are inconsistent with data obtained from clinical research on patients of epilepsy. Increased activity of SOD and GPX in the brain of epilepsy patients are reported [29]. Thus, the antioxidative response, activity of antioxidants and expression pattern changes of reduction enzymes have not been fully understood probably because of complexity of antioxidant mechanism, different brain samples and models used for seizures. In this study, the antioxidative response was first measured by examining the protein and mRNA expression levels of TRX1, TXNL1 and TXNRD1, which facilitate the antioxidative response. The results indicated that the up-regulation of these enzymes may play a protect role in the cortex during seizures while other antioxidant system is suppressed, such as GSH. In the cortex, the mRNA expression levels of these enzymes were only increased 20–30% while the protein expression levels of these enzyme were increased 2–3 fold. This phenomenon indicated that the protein expression regulation mechanism is mainly at the translational level, which is consistent with the hypothesis that a rapid antioxidative response is needed for adapting to fast oxidative stress induced by PTZ kindling. This antioxidative response may ameliorate oxidative stress in the cortex. But brain regions that absence of this protection mechanism may be more vulnerable to oxidative stress, such as hippocampus. Further studies using conditional knockout mice of these antioxidant proteins are needed to further address the role of oxidative stress in the mouse brain.

Considering the benefit of using antioxidants as the adjunct antiepileptic therapy, this study provided novel targets for developing new drugs to change redox status of the brain, probably not only in epilepsy but also in other neurological diseases that with oxidative stress, such as Alzheimer’s disease.

## Materials and methods

### Animal model preparation

The protocol and experimental design for all mice experiments was approved by the Animal Care Committee of Xuzhou Medical University. Male C57BL/6 mice (aged 10 to 12 weeks, body weight 25–30 g) were used for generating acute and chronic repetitive seizure model. The mice were bred and maintained by lab animal center of Xuzhou Medical University. Total 15 mice were used for the whole experiment. Three normal mice were sacrificed by cervical separation for collecting control brain tissues. Proteins and mRNA extracted from these brain tissues were used as control protein or control mRNA in this experiment. In the meantime, 3 mice were used to generate acute seizure model while 9 mice were used to generate chronic seizure model. The mice were used for acute seizure model were sacrificed by cervical separation within 2 hours after seizures. Every 3 mice of chronic seizure model were sacrificed by cervical separation at each time point after PTZ injection indicated in Fig 1. The mice were housed in temperature-controlled, filter ventilated system and 12h light/12h dark cycled rooms. The bedding materials, food and water were changed every three days. Lab mice care was performed according to 3Rs (replacement, reduction, refinement) guidelines agreed by IACUC of Xuzhou Medical University. No more than 5 mice were kept in each standard mouse cage. Sterile cages were used and mice were maintained in clean and filtered ventilation system made by Suzhou Fengshi Lab Equipment Company. Mice health were monitored every other day by a staff in lab animal center. There is no mouse died without euthanasia. For generating acute seizure model, 70mg/kg of PTZ was intraperitoneally injected once, full clonus of the body can be observed 1–3 min after injection. For making chronic seizure model, 35 mg/kg of PTZ intraperitoneal injection was repeated every other day between 3PM and 5PM. After each subthreshold PTZ injection, mice were observed for 30 minutes to find out whether they exhibit seizure behavior. Mice were anesthetized by 1% Pentobarbital with a dose of 0.12 ml per 25g body weight before sacrificing. The seizure intensity were scaled to 6 stages according to revised Racine’s scale method [23]. Scale stages are listed from 1 to 6. First stage: sudden behavioral arrest and/or motionless staring. Second stage: facial jerking with muzzle or muzzle and eye. Third stage: neck jerks. 4^th^ stage: Clonic seizure in a sitting position. 5th stage: convulsions including clonic and/or tonic–clonic seizures while lying on the belly and/or pure tonic seizures. 6^th^ stage: Convulsions including clonic and/or tonic–clonic seizures while lying on the side and/or wild jumping.

### EEG recording

Wild type mice were anesthetized by 1% Pentobarbital with a dose of 0.12 ml per 25g body weight. Subsequently, mice were placed in the stereotaxic apparatus and fixed by ear bars. Then, a 1 cm rostral-caudal incision through the skin on the top of the mouse head was made to expose the top surface of the skull. 3% hydrogen peroxide was used to clean and disinfect the top surface of the skull. A small amount of cyanoacrylate was put on the bottom of the 6 pore head-mount (Purchased from Pinnacle Technology Inc) for gluing the head-mount on the top of the mouse head. Then, place the head-mount on the top surface of the dry skull of the mouse until the head-mount cannot be moved. Finally, the mouse was put in separate cages to wait for PTZ injection after suturing the incision. Before peritoneal injection of PTZ, the configured preamplifier was connected to the head-mount on the head top of the mouse according to standard protocol provided by Pinnacle Technology Inc (USA) for 1 hour. The 3 channel preamplifier was further connected to computer system through wires to collect EEG signals. The data were stored in computer and were analyzed by software provided by Pinnacle Technology Inc.

### Western blot analysis

Protein samples were loaded on a polyacrylamide gel (50ug/lane) and separated by electrophoresis. Resolved proteins were transferred onto polyvinylidene difluoride (PVDF) membranes and blocked with no-fat milk for 2 hours at room temperature. The membranes were probed with affinity-purified anti-TXNRD1 (1:1000; Hangzhou HuaAn Biotechnology Co. Ltd.), anti-TXNL1 (1:1000; Abcam) or anti-TRX1 (1:1000; Abcam) antibody at 4°C overnight, followed by incubation with horseradish peroxidase (HRP)-conjugated goat anti-rabbit IgG (1:3000; Pierce). To normalize the loaded samples, affinity purified anti-glyceraldehyde-3-phosphate dehydrogenase (GAPDH) antibody (1:3000; Proteintech) was used, followed by incubation with HRP-conjugated anti-rabbit IgG (1:3000; Pierce). Images were acquired with the ChemiDoc Tm XRS+ imaging system and analyzed with imaging lab software (Bio-RAD). The density of the band of interest proteins (TXNRD1, TXNL1 or TRX1) was measured and normalized to the density of the band of GAPDH.

### RNA extraction

Total RNA was extracted from isolated brain tissues of control and seizure model mice by using Hiscript II Q RT Kit according to the manufacturer’s specifications. The yield of RNA was determined using a NanoDrop 2000 spectrophotometer (Thermo Scientific, USA), and the integrity was evaluated using agarose gel electrophoresis stained with ethidium bromide.

### Real-time quantitative RT-PCR

Quantification was performed with a two-step reaction process: reverse transcription (RT) and PCR. Each RT reaction has two steps. The first step is 0.5 μg RNA, 2 μl of 4×gDNA wiper Mix, add Nuclease-free H2O to 8 μl. Reactions were performed in a GeneAmp PCR System 9700 (Applied Biosystems, USA) for 2 min at 42°C. The second step is add 2μl of 5 × HiScript II Q RT SuperMix IIa. Reactions were performed in a GeneAmp PCR System 9700 (Applied Biosystems, USA) for 10 min at 25°C; 30 min at 50°C; 5 min at 85°C.The 10 μl RT reaction mix was then diluted × 10 in nuclease-free water and held at -20°C. Real-time PCR was performed using LightCycler 480 Ⅱ Real-time PCR Instrument (Roche, Swiss) with 10 μl PCR reaction mixture that included 1 μl of cDNA, 5 μl of 2× QuantiFast SYBR Green PCR Master Mix (Qiagen, Germany), 0.2 μl of forward primer, 0.2 μl of reverse primer and 3.6 μl of nuclease-free water. Reactions were incubated in a 384-well optical plate (Roche, Swiss) at 95°C for 5 min, followed by 40 cycles of 95°C for 10 s, 60°C for 30 s. Each sample was run in triplicate for analysis. At the end of the PCR cycles, melting curve analysis was performed to validate the specific generation of the expected PCR product. The primer sequences were designed in the laboratory and synthesized by Generay Biotech (Generay, PRC) based on the mRNA sequences obtained from the NCBI database as follows: Trx1 (Genebank NM_011660.3): 5’-CCCTTCTTCCATTCCCTCT-3’ and 5’-TCCACATCCACTTCAAGGAAC-3’, Txnl1 (Genebank NM_016792.4): 5’-TTCAAACGAGTGGTTGGCA-3’ and 5’- GCAGTAAGTCTTCCAGTGTC-3’, Txnrd1 (Genebank NM_001042513.1): 5’- GTGGCGACTTGGCTAATC-3’ and 5’- ACCAGGAGAGACACTCAC-3’, GAPDH (Genebank NM_008084): 5’- TCATCCCAGAGCTGAACG-3’ and 5’-TCATACTTGGCAGGTTTCTCC-3’. The expression levels of mRNAs were normalized to GAPDH and were calculated using the 2^-ΔΔCt^ method (Livak and Schmittgen, 2001).

### Statistics and analysis

The percentage change in the level of expression of the protein was then calculated using the formula below: ((average of normalized density for that treated with PTZ /average of normalized density for normal wild type mice) * 100%. The SEM was calculated with the following formula: (SEM of average of normalized density for that treated with PTZ/average of normalized density for normal mice) * 100%. In each time point, the western blot were repeated 3 times. The density of western blot band in each group were measured and compared using t-test. Data are expressed as mean ± SEM. The P-value is less than 5% was considered as statistically significant. The Q-PCR values of each time points were statistical analyzed by T-test. P value is less than 0.05 was considered as statistically different.

## Supporting information

S1 Dataset Excel table: Statistical results of Fluorescent RT-PCR results. The original Fluorescent RT-PCR curves and melting peaks of GAPDH, TXNL1 (Trp32), Thioredoxin and TXNRD1.
(RAR)
